# Assessment of COVID-19 death surveillance during the pandemic period in Recife

**DOI:** 10.1590/0034-7167-2023-0408

**Published:** 2025-06-27

**Authors:** Adriana Carla de Luna Ribeiro, Conceição Maria de Oliveira, Vilma Costa de Macêdo, Paulo Germano Frias, Cristine Vieira do Bonfim

**Affiliations:** I Universidade Federal de Pernambuco. Recife, Pernambuco, Brazil; II Prefeitura do Recife, Secretaria de Saúde. Recife, Pernambuco, Brazil; III Instituto de Medicina Integral Prof. Fernando Figueira. Recife, Pernambuco, Brazil; IV Fundação Joaquim Nabuco. Recife, Pernambuco, Brazil

**Keywords:** COVID-19, Death, Epidemiological Monitoring, Public Health, Pandemics, COVID-19, Muerte, Monitoreo Epidemiológico, Salud Pública, Pandemias

## Abstract

**Objective:**

To evaluate the surveillance of deaths due to COVID-19 during the pandemic in Recife, Pernambuco, between 2020 and 2021.

**Methods:**

Evaluative study of the analysis type, which analyzes the transformation of the surveillance of deaths due to COVID-19. Data collected through structured participant observation, consultation of public documents and records of the Mortality Information System.

**Results:**

89 deaths were investigated and discussed, of which 64 (71.9%) were attributed to COVID-19 as the underlying cause and 14 (15.7%) were reclassified. The investigation was carried out by telephone call and consultation of medical records. The technical group identified errors in 52 (58.4%) deaths. The online discussions and recommendations to managers had uncertain frequency.

**Final considerations:**

Death surveillance revealed improvements in the causes of death and recommended preventive measures to reduce mortality. The reassignment of professionals was observed, affecting the frequency of discussions and the implementation of recommendations. The findings reinforce the importance of initiatives that strengthen death surveillance.

## RESUMEN

**Objetivo:**

Evaluar la vigilancia de las muertes por COVID-19 durante la pandemia en Recife, Pernambuco, entre 2020 y 2021.

**Metodo:**

Estudio evaluativo que analiza la transformación de la vigilancia de las muertes por COVID-19. Datos recolectados a través de observación participante y estructurada, consulta de documentos públicos y registros del Sistema de Información de Mortalidad.

**Resultados:**

Se investigaron y discutieron 89 muertes, 64 (71,9%) fueron atribuidas a la COVID-19 como causa subyacente y 14 (15,7%) fueron reclasificadas. La investigación se realizó vía teléfono y por la consulta de historias clínicas. El grupo técnico identificó errores en 52 (58,4%) defunciones. Las discusiones en línea y las recomendaciones a los gerentes tenían una frecuencia incierta.

**Conclusiones:**

La vigilancia de la muerte reveló la mejora en las causas de muerte y recomendó medidas preventivas para reducir la mortalidad. Se observó la reubicación de profesionales, afectando la frecuencia de las discusiones y el envío de recomendaciones. Los hallazgos refuerzan la importancia de las iniciativas que fortalezcan la vigilancia de las muertes.

## INTRODUCTION

The COVID-19 pandemic has certainly been the greatest health challenge of this century, due to its exponential spread, its ability to overwhelm health systems and cause deaths and socioeconomic repercussions in all countries^(1)^. In Brazil, between 2020 and 2021, the incidence of the disease was 10,605 cases per 100,000 inhabitants, the mortality rate was 294 deaths per 100,000 inhabitants and the lethality rate was 2.7%, while in Recife the incidence was 9,814 cases per 100,000 inhabitants, the mortality rate was 345.93 deaths per 100,000 inhabitants and the lethality rate was 3.5%^(2)^. To face this pandemic, healthcare and epidemiological surveillance needed to adapt quickly in order to increase the supply of beds, implement accurate diagnostic testing, trace contacts, monitor cases and make quality and timely data available^(3)^.

Surveillance of deaths due to COVID-19 includes investigation with family members and medical records, review of death certificates (DC) and analysis of information^(4)^. This surveillance was crucial to understanding the factors that contributed to the occurrence of many deaths, the impact of SARS-CoV-2 variants, vaccination and, above all, recommending preventive measures and avoiding new deaths^(5)^.

In Brazil, the spread of COVID-19 was monitored using data from the Influenza Epidemiological Surveillance Information System (Sivep-Gripe) and DCs^(6)^. In Recife (PE), the surveillance of deaths due to COVID-19, an innovative intervention for qualifying causes of death during the pandemic, began motivated by the lack of clinical and preventive evidence about the disease, supported by similar previous initiatives carried out for other types of death and by international and national experiences, having been adjusted to local needs^(3,7)^.

Knowledge about COVID-19 death surveillance contributes to its improvement, the indication of preventive actions and clinical practice^(7)^. Studies on death surveillance present results, but do not describe the beginning and progress of the stages, especially during a Public Health Emergency of International Concern (PHEIC)^(8,9)^. To understand the changes of a new intervention, with little control over external and organizational contexts, in the face of dynamics with a procedural focus, some methodological approaches were proposed, among them, the analysis of implementation with an emphasis on the transformation of the intervention^(10)^.

## OBJECTIVE

To assess the surveillance of deaths due to COVID-19 during the pandemic period in Recife, Pernambuco, between 2020 and 2021.

## METHODS

### Ethical aspects

The study was conducted in accordance with national and international ethics guidelines and approved by the Research Ethics Committee of the Federal University of Pernambuco.

### Type of study and theoretical-methodological framework

Evaluative research using typology proposed by Champagne *et al*.^(10)^ for analyzing the transformation of the intervention, classified as type 1-a in implementation analyses. This type of evaluation aims to explain how the intervention adapts to its context, how the change in form, scope and nature occurs over the course of its development, in its design, implementation and routinization ^(10)^. The intervention under analysis was the surveillance of deaths due to COVID-19 among people residing in Recife, in the years of 2020 and 2021. The case study strategy was used to approach the object of the evaluation and express how death surveillance changed during the period from the beginning of PHEIC due to COVID-19, until 2021, based on the interaction with the national and local contexts. The SRQR (Standards for Reporting Qualitative Research) protocol was used for the research stages that were included^(11)^.

### Methodological procedures

A logical model of the intervention was developed, with an explanation of each component of the surveillance of deaths due to COVID-19, taking as a reference a previous model developed in a death surveillance study^(12)^. The logical model is a tool used to present the intervention in a systematic way, which describes relationships between the components, their operationalization and the expected results^(13)^. To construct the logical model, considering the adjustments made due to the PHEIC, manuals, guidelines and technical notes from the World Health Organization (WHO), the Ministry of Health (MH), and from the Health Secretariat of Recife and Pernambuco were used. The changes that occurred during the study period, the facilitators and obstacles to development and the implications for the results from the perspective of the researchers who worked directly on the strategy were described.

### Study scenario

The intervention took place in the city of Recife, with an urbanized territorial area of 218.5 km^2^, population of 1,488,920 inhabitants for 2022^(14)^, divided into 94 neighborhoods, grouped into six Political-Administrative Regions and eight Health Districts^(15)^. As of April 2020, the municipality made seven field hospitals available, with 1,093 beds (352 ICU and 741 ward beds) to care for patients with suspected or confirmed cases of the disease^(16)^.

On March 12, 2020, the first imported cases were confirmed and two days later, local transmission of COVID-19 was established in Recife. At this stage, municipal surveillance monitored all notified individuals^(16)^. On March 17, 2020^(16)^, with the establishment of community transmission and an increase in the number of infected people, monitoring became limited to hospitalized people, through telephone calls or e-mails to family members and/or hospital epidemiological surveillance (HES). In view of the growing demand, the municipal epidemiological surveillance service has been gradually strengthened. The number of professionals involved in the surveillance of respiratory viruses increased from three (two technicians and one coordinator) to 45 people, of which 36 were nurses.

### Data collection and organization

The results described come from structured participant observation by researchers who worked on the design, development and strategy implementation. Four researchers are PhDs with over 20 years of experience in public health, surveillance and public health emergencies, and one researcher has a master’s degree, with a similar experience but less time. Three researchers work at the municipal health department, one at a public university and one at a social research foundation. Regarding those with ties to the department, one works in the health care sector and the others in epidemiological and mortality surveillance, which, on the one hand, facilitates access to strategic information for the reconstruction of the intervention and, on the other, can influence the description of occurrences and minimization of obstacles to pandemic management.

The retrospective approach may imply loss of information, especially due to the lack of documentation of micro work processes associated with possible memory bias. To minimize these effects, the product of this work was submitted to the technicians involved in surveillance for confirmation, or not, of the findings.

In addition, an analysis of public documents related to this surveillance has been carried out, available on the official websites of the World Health Organization (WHO), the Ministry of Health, and the Health Departments of Recife and Pernambuco, such as international guidelines, technical notes, manuals for coding the causes of death, handling of bodies during the pandemic, and the contingency plan for COVID-19 (Chart 1). The documents were accessed from August 2022 to July 2023.

**Chart 1:** List of official documents on COVID-19 consulted from the World Health Organization, the Ministry of Health, the Health Department of Pernambuco and Recife, Brazil, between 2020 and 2021

Document	Content
International guidelines for the certification and coding of COVID-19 as a cause of death, World Health Organization, April 20, 2020^(17)^.	It presents the definition of causes of death, guidelines for the certification of COVID-19 as a cause of death and in terms of mortality.
Provisional recommendations on the handling of bodies in the context of COVID-19, World Health Organization, April 7, 2020^(18)^.	It deals with the handling of bodies in the context of COVID-19, autopsy, environmental cleaning and personal protective equipment.
Manual for handling bodies in the context of the Sars-CoV-2 coronavirus - COVID-19, version 1 and 2, Ministry of Health, March and November, 2020^(19-20)^.	Addresses recommendations for conducting wakes, burials, autopsies and issuing death certificates (DC) in the context of COVID-19.
Information note no. 02 for coding causes of death for suspected or confirmed deaths from COVID-19, March 2020, Pernambuco Health Department^(21)^.	It covers the recommendations for filling in the causes of death in PART I of the DC, for suspected deaths or deaths with a positive laboratory result for COVID-19.
Technical note DG-IAEVE of SEVS/Pernambuco Health Department No. 04 of March 25, 2020, on the handling of bodies in the context of infection by the new coronavirus - COVID-19^(22)^.	Provides for the forwarding of bodies to the Death Certification Review Service (DCRS), Legal Medical Institute (LMI), funeral homes, holding of wakes, burials and guidance to health services on the collection of material for examinations and preparation of bodies in the context of COVID-19.
Technical note from SEVS of the Pernambuco Health Department No. 7 of April 14, 2020, on epidemiological and laboratory surveillance in the COVID-19 pandemic^(23)^.	Updates health professionals in Pernambuco on notification, definition of suspected, confirmed and inconclusive cases of influenza-like syndrome (IS) and severe acute respiratory syndrome (SARS), collection, packaging and transportation of samples and issuing of DC.
Information note No. 07 of May 5, 2021 from the Epidemiological Surveillance Management of the Recife Health Department^(24)^.	Provides the objectives, characteristics, flow, activities carried out and stages of surveillance of deaths due to COVID-19.
Municipal COVID-19 Contingency Plan of the Recife Health Department, March 10, 2020 ^(16)^.	Describes the response levels and proposed activities, list of technical areas and those responsible, assistance flow of suspected cases in primary health care and in SAMU.

*Key: DC= death certificate; ES= epidemiological surveillance; HES= hospital epidemiological surveillance; SRAS= severe acute respiratory syndrome; MIS= mortality information system; SIVEP-Flu= influenza epidemiological surveillance system; Notifica PE= information system for recording serious cases of COVID-19 used in Pernambuco.*

### Work stages

The research began with the development of the logical model, its components and explanation of the flows. Subsequently, the collection techniques and data organization were defined, the timeline was constructed and the main changes in the components of death surveillance were identified, considering national and local contexts. Finally, the findings of the investigation of deaths in Recife (PE), in 2020 and 2021, were presented.

### Data analysis

A link was made through automatic research of the variables number of the DC and patient name from the Death Spreadsheet for SARS and COVID-19 with the Mortality Information System (MIS) database for the investigated and discussed deaths that occurred in 2020 and 2021. For each DC paired with the Death Spreadsheet line, the date of birth and the mother’s name were manually checked to validate the true pair. For deaths that were not located automatically, after manual research, all were located in the MIS and the causes of death were analyzed using Microsoft Excel. Access to secondary data from the MIS and the Death Spreadsheet due to SARS and COVID-19, with nominal information on cases that evolved to death, was authorized by the Research Ethics Committee of the Federal University of Pernambuco.

## RESULTS

**Figure 1:**
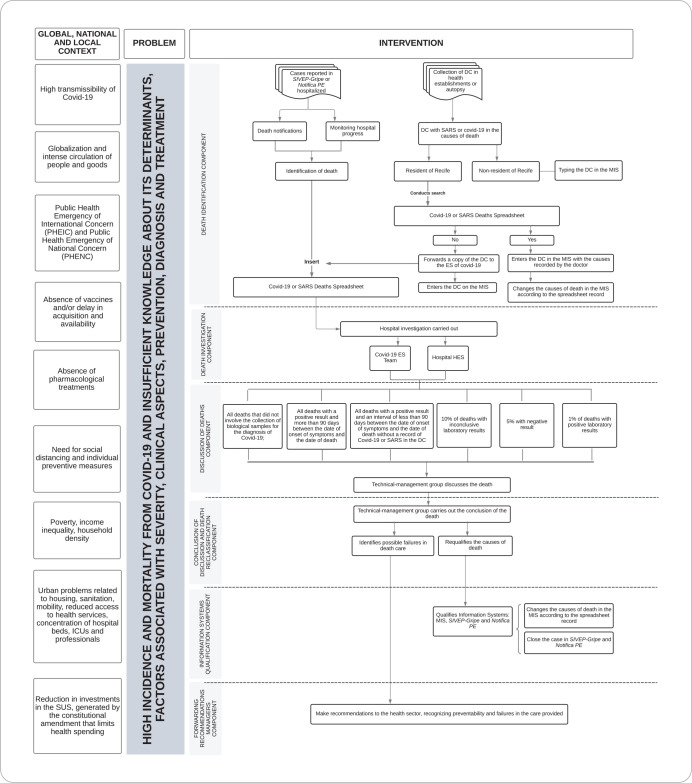
Logical model, operational components and activities of COVID-19 death surveillance in Recife, Pernambuco, Brazil, in 2020 and 2021

The surveillance of deaths due to COVID-19 in Recife was dynamically mediated based on the characteristics of this public health emergency and the socioeconomic and health care determinants in the global contexts of Brazil and Recife. The development of death surveillance was gradually ordered, due to the limitations imposed by current health measures and the organization of municipal surveillance, based on its components (Figure 1).

- Identification of deaths due to SARS or COVID-19.
- Epidemiological investigation of death due to SARS or COVID-19.
- Discussion of deaths from SARS or COVID-19.
- Conclusion of the discussion and reclassification of deaths due to SARS or COVID-19.
- Qualification of health information systems.
- Forwarding recommendations to managers.

The initial component, identification of deaths due to SARS or COVID-19, aimed to capture suspected or confirmed deaths of residents of Recife, detected through notification or monitoring of the evolution of hospitalized cases or in the DC, when described as SARS or COVID-19 in the causes of death. The collection of DCs in health facilities, especially with the inauguration of seven field hospitals in the city of Recife, began to be carried out more frequently, up to three times a week, when compared to the routine before the pandemic.

Between 2020 and 2021, 9,703 deaths were identified and entered into the “Deaths Spreadsheet due to SARS and COVID-19” by the municipal epidemiological surveillance team. The registration as SARS of suspected COVID-19 infected deaths without laboratory confirmation followed the recommendation of the Pernambuco Health Department.

The subsequent component, investigation of deaths due to SARS or COVID-19, sought to confirm the address of residence and identify the events that contributed to the death. Due to health restrictions, these actions were carried out by means of a telephone call made by the municipal epidemiological surveillance team in 2020. The aim was to identify the correct address of residence, provide assistance, supplement information, provide guidance to the family member or contact of the case that evolved to death on health measures, schedule an examination and direct them to psychological care when necessary.

The results of tests that complemented the investigation were obtained by consulting the Laboratory Environment Manager (LEM), a system for public health laboratories. If the results were not found in the initial search, the results were requested from the HES teams or private laboratories.

With the organization of hospital flows, at the end of 2020, deaths occurring in health services with HES began to be adopted as an inclusion criterion. The Pernambuco Health Department prepared an epidemiological investigation form and established the investigation of deaths by consulting medical records through HES. The investigation form consisted of variables grouped into case identification information; personal history; initial clinical condition and upon hospitalization; diagnosis upon admission; laboratory and tomographic findings; complications; pre-hospital transport; DC; classification and death closure criteria.

At this stage, deaths could have outcomes such as: confirmed or ruled out for COVID-19, unspecified SARS, other respiratory viruses, other causes of death or being a resident of another municipality. Among the total number of deaths recorded, 7.9% (764) corresponded to residents of other municipalities.

The discussion of deaths, the third component, was intended to identify possible failures in care provided by health services, recognizing their preventability and contributing to the closure of deaths with mention of SARS or COVID-19. This phase has an educational, reflective and proactive function, and is not coercive or punitive.

In 2020, due to the limited availability of specific laboratory tests, all deaths that did not involve the collection of biological samples were selected for discussion. Starting in the first half of 2021, the criteria for selecting deaths for discussion became: deaths with a positive test result for COVID-19 and more than 90 days between the date of the beginning of symptoms and the date of death, or those with an interval of less than 90 days without a record of COVID-19 or SARS in the DC; 10% of deaths with inconclusive laboratory results and 5% with negative results and 1% of deaths with positive laboratory results. However, it was observed that, as a priority, deaths with available hospital investigations carried out by HES teams were discussed.

The discussions were held by the technical management group for death surveillance, composed of professionals from municipal epidemiological surveillance, from the technical area of COVID-19, mortality, and by doctors from the health network, with at least one infectious disease specialist. The meetings were held weekly, and up to five deaths were discussed, always online or in a hybrid format, at the surveillance headquarters. The frequency of the meetings changed due to the reallocation of death surveillance professionals to other activities according to the waves of case occurrence, which occurred between October 2020 and January 2021 and between March and June of 2021. A total of 89 deaths that occurred between March 2020 and December 2021 were investigated and discussed in a technical group. Of these, 60 (67.4%) deaths were confirmed for COVID-19, 55 (61.8%) by laboratory criteria, 5 (5.6%) by epidemiological clinicians, and 29 (32.6%) were discarded.

The fourth component, conclusion of the discussion and reclassification of deaths due to SARS or COVID-19, sought to identify possible failures in care associated with deaths and defined intervention recommendations to be forwarded. Deaths were finalized with the information obtained in the investigation as: confirmed by laboratory, clinically or by epidemiological criteria and discarded.

In the first year of the pandemic, for the purposes of recording in the case notification information systems, Notifica PE and Sivep-Flu, a laboratory result confirming or ruling out death was required. Although technical notes included the possibility of closing a COVID-19 case based on clinical epidemiological criteria, the unspecified SARS classification was used for cases without laboratory sample collection.

Regarding the registration of deaths from COVID-19, in March 2020, the Ministry of Health and the Health Department of Pernambuco recommended the use of code U04.9 (SRAS) for deaths with suspected COVID-19 infection without laboratory diagnosis. For those with a positive laboratory result for COVID-19, part I of the DC was completed with code B34.2 (coronavirus infection - COVID-19) and for deaths with acute respiratory disease due to COVID-19, the marker, code U04.9, was used on the same line as code B34.2.

The purpose of the Health Information Systems (HIS) qualification component was to correct official statistics. After the death was confirmed, the causes of death in the MIS were requalified in accordance with the recommendations of the World Health Organization in laboratory-confirmed code B34.2 + U07.1 of the International Statistical Classification of Diseases and Related Health Problems (ICD-10), clinical-epidemiologically confirmed code ICD-10 B34.2 + U07.2 and discarded using the code marker ICD-10 U04.9^(17)^. The coding of causes of death before and after the discussions was carried out by trained, highly experienced, underlying cause coders working at the Municipal Health Department.

The ICD-10 codes, mainly in Part II of the DC, referring to pre-existing morbidities and not directly related to death, followed by line B, underwent modifications. The underlying cause presented 34.8% of change after the discussion and qualification of deaths (Table 1).

**Table 1:** Distribution of the number and proportion of deaths with maintenance or change of the ICD-10 code after the discussion, according to the sequence of causes of death. Recife, Pernambuco, Brazil, 2020-2021

		Deaths with maintenance of the ICD-10 Code		Deaths with changes in the ICD-10 Code	
		n°	%	n°	%
PART I	Line A	49	55.1	40	44.9
	Line B	38	42.7	51	57.3
	Line C	50	56.2	39	43.8
	Line D	74	83.1	15	16.9
PART II		25	28.1	64	71.9
ROOT CAUSE		58	65.2	31	34.8

*Note: ICD-10= International Statistical Classification of Diseases and Related Health Problems.*

Regarding exclusively the analysis of the underlying cause of death, of the 89 deaths investigated and discussed in the period between 2020 and 2021, 64 (71.9%) had COVID-19 as the underlying cause. After the conclusion of the case, 14 (15.7%) deaths were reclassified to other causes. For those deaths (25; 28.1%) that did not have COVID-19 as the underlying cause, six (6.7%) underwent the qualification and were attributed to COVID-19 as the determining cause of death.

The last component, forwarding recommendations to managers, aimed to send reports of investigated and discussed deaths to those responsible for the sectors of surveillance, assistance, regulation and planning in municipal health. After the discussion, a report was prepared for each death and forwarded to managers for decision-making, containing: the problems identified and the reclassification of the causes of death. This procedure was the most scarce, carried out for deaths discussed in the first meetings.

Of the 89 deaths discussed, 52 (58.4%) had failures identified by the technical group during the illness and death process, while for 37 (41.5%) deaths, there was no such indication. For the deaths in which failures were identified, their respective recommendations were grouped and related to the diagnosis, of an individual, care, registration and notification nature (Chart 2).

**Chart 2:** Main flaws and recommendations identified in the discussion of deaths. Recife, Pernambuco, Brazil, 2020-2021

Type	Flaw	Recommendation
Diagnosis	Failure to request and/or perform clinical laboratory and imaging tests Late performance of Reverse Transcriptase Polymerase Chain Reaction (RT-PCR) test for COVID-19	Timely performance of the RT-PCR test for COVID-19 Performance of imaging tests to aid in diagnosis
Individual	Late seeking health care Lack of complete vaccination schedule for COVID-19	Optimization of the link between the primary care team and users in the area Promotion of educational activities on warning signs of the severity of the disease Active search to understand the lack of a vaccination schedule for COVID-19
Assistance	Failure to perform cardiological assessment Improper transfer to a referral hospital for COVID-19 of a patient with a negative RT-PCR result Lack of general nursing care Lack of sedative and bronchodilator medications Lack of or malfunctioning hospital equipment (mechanical ventilators, infusion pumps, oxygen sources, heaters) Failure to access health services (lack of ICU beds) Iatrogenesis related to medical care Infection related to health care (COVID-19, fungal urinary tract infection) Failure to monitor and ensure patient safety	Presence of nursing supervision in hospital services Provision of equipment and medicines for patient care Development of protocols and training for good practices in invasive procedures Assessment and reorganization of care flows (clean area and contaminated area) Application of the patient safety protocol in services Application of the nosocomial infection prevention protocol in services Availability of equipment maintenance team Critical and specialized assessment of the clinical condition for timely transfer
Registration and Notification	Failure to provide details or lack of in-hospital and patient transfer records Discrepancies in information between different record sources	Detailed recording in hospital records and transfer summaries Complete completion of the SARS death investigation form

*Note: ICU = Intensive Care Unit; RT-PCR= reverse transcriptase reaction followed by polymerase chain reaction; SRAS= severe acute respiratory syndrome.*

The main obstacles to implementing the components of death surveillance were related to the lack of clinical evidence about the disease, the limited supply of diagnostic tests, the absence of ICD-10 codes for COVID-19, restricted access to health services and the handling of medical records at the beginning of the pandemic. Likewise, the reallocation of professionals to activities unrelated to death surveillance was another obstacle to its full development (Chart 3).

**Chart 3:** Facilitators and obstacles according to the component of COVID-19 death surveillance. Recife, Pernambuco, Brazil, 2020-2021

Component	Facilitators	Obstacles
Identification of deaths from SARS or COVID-19	Monitoring the evolution of hospitalized cases, identifying deaths	Clinical evidence on limited disease early in the pandemic
Epidemiological investigation of death from SARS or COVID-19	Experience of the municipal and hospital epidemiological surveillance team in investigating cases and deaths from notifiable diseases	Restriction on access to health services and handling of medical records, at the beginning of the pandemic
Discussion of deaths from SARS or COVID-19	*Availability of videoconferencing equipment for holding online death discussion meetings*	Limited availability of diagnostic tests Reallocation of death surveillance professionals
Qualification of health information systems	Qualification of information systems previously incorporated into the routine of municipal epidemiological surveillance	Change of case of disease definition Absence of ICD-10 codes for COVID-19

*Note: SRAS= severe acute respiratory syndrome. ICD-10= International Statistical Classification of Diseases and Related Health Problems.*

## DISCUSSION

The analysis of the transformation of the surveillance of deaths due to COVID-19 during the health emergency showed that the intervention was developed to prevent new deaths resulting from similar situations, understand the chain of events and qualify the MIS. It started from the notification of the first deaths by the health services to the municipal epidemiological surveillance, involving the sectors responsible for the epidemiological surveillance of communicable diseases, the Center for Strategic Information on Health Surveillance (Cievs) and for mortality.

All components were gradually operationalized by the epidemiological surveillance of Recife. Initially, the death surveillance was developed to retrieve information (address, clinical history and laboratory results), qualify the MIS and provide guidance to contacts and/or family members of people who died. Later, it increased, with the performance of hospital investigation, discussion of deaths, identification of problems and proposals of recommendations to managers.

Studies show that there was a social determination in the incidence and mortality from COVID-19. The expansion of the pandemic occurred in areas of greater vulnerability, associated with the economic crisis, socioeconomic factors, inadequate housing conditions, a higher proportion of people living in overcrowded housing and a higher percentage of people without education^(25)^.

The recording of deaths carried out by epidemiological surveillance, in addition to COVID-19, included SARS among the causes recorded in the DC, to cover cases without laboratory confirmation. Inclusion aligned with the World Health Organization’s recommendation for monitoring deaths related to non-specific respiratory causes, linked to little certainty about the disease and variability in the supply of tests ^(26,27)^. The underreporting of deaths was highlighted in a study on the African continent, of post-mortem surveillance, with RT-PCR performed on the deceased^(8)^.

Confirmation of the residential address, part of the initial investigation of the cases, allowed this information to be rectified, bringing the real number of deaths of residents closer. It is known that the completeness of the investigation records provides information, directs interventions and investments^(28)^. Limitations are observed in the investigation of infant and fetal deaths, due to the difficulty in validating the residential address of the deceased or the mother^(29)^.

The results of specific tests were retrieved from various sources during the investigation of deaths. The identification of infected people allows their timely and appropriate isolation^(30)^. However, in the beginning of the pandemic, the scope of testing was limited, especially in low-income or developing countries, due to the high cost of supplies and labor^(30)^. In Recife, the State Health Department was responsible for the inputs for collecting and processing the sample, leaving the municipality to perform the collection and make the results available to the patient.

Deaths that occurred in health units without a HES were not chosen for discussion, due to health measures that restricted access to health services and medical records. The HES plays an important role in the reporting unit, integrating information systems and raising awareness among professionals, during responses to other public health emergencies of national and international interest, such as the influenza A HIN1 pandemic and the Zika virus-related microcephaly pandemic^(31)^. The role of nurses in the HES and in municipal epidemiological surveillance stands out. In addition to providing clinical care within the context of the pandemic, this professional has the qualifications, technical and legal competence to develop research and communication^(32)^.

Due to the dynamic nature of the context of an Espii, the frequency of discussion meetings was modified and recommendations to managers were not forwarded for all deaths. Despite the hiring and mobility of professionals, especially nurses, from other sectors for epidemiological surveillance, there was internal repositioning for activities other than those related to death surveillance. The work of these professionals was redirected to providing guidance to family members and correcting the causes of death. The spread of fake news about the origin, symptoms and medication for COVID-19, fueled in Brazil by the federal government’s denial of the relevance of the pandemic and social distancing measures, led to the need for ongoing guidance to the population^(33,34)^.

Even though, with the redirection of municipal epidemiological surveillance teams, the qualification of the causes of death was observed, with the modification of the ICD-10 code, of Part II of the DC of the investigated and discussed deaths. This finding is supported by the study on the quality of records of deaths due to COVID-19 between March 2020 and December 2021, which identified the maintenance of the quality of MIS information in Recife through the analysis of completeness, during the pandemic, when compared to previous years^(35)^.

The contribution of death surveillance to the classification of causes of death, including COVID-19 as the underlying cause, corroborates a study carried out in the first year of the pandemic in African countries, in which the analysis of deaths improved estimates of mortality from the disease^(8)^. In New York City, the inspection of death certificates contributed to more realistic numbers of COVID-19 deaths in 2020^(26)^. In Ireland, in 2022, death surveillance helped to properly record the cause of death related to COVID-19^(9)^.

The surveillance of deaths due to COVID-19, instituted during a PHEIC, remains active in Recife, with adaptation of the criteria for selecting deaths to be discussed, given the endemic scenario of COVID-19. In addition to COVID-19, infant, maternal and tuberculosis deaths are subject to death surveillance in Recife^(7,35-37)^.

The study demonstrated the importance of death surveillance in Recife, using it as a strategy to reduce underreporting, improve mortality statistics and recommend measures for promotion, prevention, diagnosis and treatment to avoid new deaths. Although the experience is recent and needs improvement, based on the lessons learned during the PHEIC, we believe it has the potential for national use, since it was based on the established death surveillance systems in Brazil, such as infant, fetal and maternal.

The findings reinforce the importance of initiatives that strengthen and promote the advancement of death surveillance. The development of COVID-19 death surveillance has been limited by the difficulty of accessing diagnostic tests, underreporting, peaks in the occurrence of the disease and the reallocation of professionals to other activities.

### Study Limitations

The study is limited by the lack of documents or other types of information sources that could identify municipal epidemiological surveillance flows and record precise dates of the changes in conduct and routines for death surveillance. Moreover, the evaluation of the intervention’s transformation, when using a retrospective approach, is subject to memory bias, which may result in loss of information. On the other hand, when based on a dynamic conception of COVID-19 death surveillance during a pandemic period, the boundary between the intervention and its context is tenuous, an aspect that may contribute to submerging relevant contextual aspects not explored in depth in the design. In this situation, it is noteworthy that the number of important contextual variables far exceeded the observation points.

### Contributions to the areas of Nursing, Health and Public Policies

Surveillance of deaths due to COVID-19 has been developed during the largest PHEIC, a challenging and uncertain scenario. Despite the high turnover of professionals, exacerbated by the pandemic and resulting from frequent job reassignments, death surveillance contributed to improving the quality of information, adopting preventive measures and reducing mortality. Also, it plays a training role for nurses and other professionals involved in this surveillance in health departments and HES teams. Furthermore, few studies analyzing the transformation of the intervention during the changes imposed by the PHEIC are available, and its contribution to the surveillance of deaths due to COVID-19 demonstrates its relevance.

## FINAL CONSIDERATIONS

The analysis of the transformation of the surveillance of deaths due to COVID-19 showed the adaptations of the intervention components in the context in which it was developed, which were imposed by the public health emergency, and contributed to the improvement in the specification of the causes of death. The reassignment of professionals affected the frequency of discussions on deaths and the forwarding of recommendations to managers regarding the problems identified. Although carried out during the Public Health Emergency of International Concern, the surveillance of deaths due to COVID-19 proved to be an important strategy, contributing to the qualification of information, the planning and organization of assistance and surveillance flows, as well as the recommendation of preventive measures to reduce mortality.
