# Supplementary material for: Assessment of COVID-19 death surveillance during the pandemic period in Recife
Source: Rev Bras Enferm. 2025 Jun 27;78(2):e20230408. doi: 10.1590/0034-7167-2023-0408 (PMC12208683; doi:10.1590/0034-7167-2023-0408)
Supplement: Translation

# Translation: Avaliação da vigilância do óbito por COVID-19 durante o período pandêmico no Recife

**DOI:** 10.1590/0034-7167-2023-0408pt

**Authors:** Adriana Carla de Luna Ribeiro, Conceição Maria de Oliveira, Vilma Costa de Macêdo, Paulo Germano Frias, Cristine Vieira do Bonfim

**Affiliations:** I Universidade Federal de Pernambuco. Recife, Pernambuco, Brasil; II Prefeitura do Recife, Secretaria de Saúde. Recife, Pernambuco, Brasil; III Instituto de Medicina Integral Prof. Fernando Figueira. Recife, Pernambuco, Brasil; IV Fundação Joaquim Nabuco. Recife, Pernambuco, Brasil

**Keywords:** COVID-19, Morte, Monitoramento Epidemiológico, Saúde Pública, Pandemias

## Abstract

**Objetivo:**

Avaliar a vigilância do óbito por COVID-19 durante a pandemia no Recife, Pernambuco, entre 2020 e 2021.

**Métodos:**

Estudo avaliativo do tipo análise da transformação da vigilância do óbito por COVID-19. Dados coletados por observação participante e estruturada, consulta aos documentos públicos e registros do Sistema de Informações sobre Mortalidade.

**Resultados:**

Foram investigados e discutidos 89 óbitos, 64 (71,9%) atribuem-se à COVID-19 a causa básica e 14 (15,7%) foram reclassificados. A investigação foi realizada por contato telefônico e consulta aos prontuários. O grupo técnico identificou falhas em 52 (58,4%) óbitos. As discussões *on-line* e recomendações aos gestores tinham periodicidade incerta.

**Conclusões:**

A vigilância do óbito elucidou a melhoria das causas de morte e recomendou medidas preventivas para a redução da mortalidade. Observou-se o remanejamento de profissionais afetando a periodicidade das discussões e o encaminhamento das recomendações. Os achados reforçam a importância de iniciativas que fortaleçam a vigilância de óbito.

## INTRODUÇÃO

A pandemia pela COVID-19 foi certamente o maior desafio sanitário deste século, em decorrência da sua disseminação exponencial, capacidade de esgotamento dos sistemas de saúde e de provocar óbitos e repercussões socioeconômicas em todos os países^(1)^. No Brasil, entre 2020 e 2021, a incidência da doença foi de 10.605 casos por 100 mil hab., a taxa de mortalidade, 294 óbitos por 100 mil hab. e a letalidade foi de 2,7%, enquanto no Recife a incidência foi de 9.814 casos por 100 mil hab., a mortalidade, 345,93 óbitos por 100 mil hab. e a letalidade, de 3,5%^(2)^. Para o enfrentamento dessa pandemia, a assistência à saúde e a vigilância epidemiológica precisaram se adaptar rapidamente, para aumentar a oferta de leitos, implementar teste diagnóstico preciso, rastrear contatos, acompanhar casos e disponibilizar dados de qualidade e oportunos^(3)^.

A vigilância do óbito por COVID-19 engloba a investigação com familiares e em prontuários, revisão de declarações de óbitos (DO) e análise de informações^(4)^. Essa vigilância foi decisiva para compreender os fatores que contribuíram para a ocorrência das muitas mortes, o impacto das variantes do SARS-CoV-2, da vacinação e sobretudo recomendar medidas preventivas e evitar novos óbitos^(5)^.

No Brasil, o acompanhamento da disseminação da COVID-19 ocorreu a partir de dados do Sistema de Informação da Vigilância Epidemiológica da Gripe (Sivep-Gripe) e DO^(6)^. No Recife (PE), a vigilância do óbito por COVID-19, intervenção inovadora por qualificar causas de morte durante a vigência da pandemia, iniciou-se motivada pelas poucas evidências clínicas e preventivas sobre a doença, amparando-se em iniciativas prévias similares realizadas para outros tipos de óbito e em experiências internacionais e nacionais, ajustadas às necessidades locais^(3,7)^.

O conhecimento sobre a vigilância do óbito por COVID-19 contribui para o seu aperfeiçoamento, a indicação de ações preventivas e para a prática clínica^(7)^. Estudos sobre a vigilância do óbito apresentam resultados, mas não descrevem o início e progresso das etapas, sobretudo durante uma Emergência em Saúde Pública de Interesse Internacional (Espii)^(8,9)^. Para entendimento das mudanças de uma intervenção nova, com pouco controle sobre os contextos externos e organizacionais, diante de dinâmica com foco processual, algumas abordagens metodológicas foram propostas, entre elas a análise de implantação com ênfase na transformação da intervenção^(10)^.

## OBJETIVO

Avaliar a vigilância do óbito por COVID-19 durante o período pandêmico no Recife, Pernambuco, entre 2020 e 2021.

## MÉTODOS

### Aspectos éticos

O estudo foi conduzido de acordo com as diretrizes de ética nacionais e internacionais e aprovado pelo Comitê de Ética em Pesquisa da Universidade Federal de Pernambuco.

### Tipo de estudo e referencial teórico-metodológico

Pesquisa avaliativa utilizando tipologia proposta por Champagne et al.^(10)^ para análise da transformação da intervenção, classificada como do tipo 1-a nas análises de implantação. Esse tipo de avaliação visa explicar a maneira como a intervenção se adapta a seu contexto, como ocorre a mudança de forma, de envergadura e de natureza, durante o decorrer do tempo do seu desenvolvimento, na concepção, na implantação e na rotinização^(10)^. A intervenção sob análise foi a vigilância do óbito por COVID-19 de pessoas residentes em Recife, nos anos 2020 e 2021. Utilizou como estratégia o estudo de caso, para aproximar do objeto da avaliação e expressar como a vigilância do óbito se modificou no decorrer do período de início da Espii pela COVID-19 até 2021, a partir da interação com o contexto nacional e local. Foi utilizado, para as etapas da pesquisa que se enquadravam, o protocolo SRQR (Standards for Reporting Qualitative Research)^(11)^.

### Procedimentos metodológicos

Elaborou-se um modelo lógico da intervenção, com a explanação de cada componente da vigilância do óbito por COVID-19, tomando como referência um modelo prévio elaborado em um estudo de vigilância de óbito^(12)^. O modelo lógico é uma ferramenta utilizada para apresentar a intervenção de forma sistematizada, que descreve relações entre os componentes, sua operacionalização e os resultados esperados^(13)^. Para a construção do modelo lógico, considerando os ajustes devidos à Espii, foram utilizados manuais, diretrizes e notas técnicas da Organização Mundial da Saúde (OMS), do Ministério da Saúde (MS), da Secretaria de Saúde do Recife e de Pernambuco. Foram descritas as mudanças ocorridas no período do estudo, os facilitadores e obstáculos ao desenvolvimento e as implicações nos resultados na perspectiva dos pesquisadores que atuaram diretamente na estratégia.

### Cenário do estudo

A intervenção ocorreu na cidade de Recife, com área territorial de 218,5 km^2^ urbanizada, população de 1.488.920 habitantes para 2022^(14)^, dividida em 94 bairros, aglutinados em seis Regiões Político-Administrativas e em oito Distritos Sanitários^(15)^. A partir de abril de 2020, o município disponibilizou sete hospitais de campanha, com 1.093 leitos (352 de UTI e 741 de enfermaria) para atendimento a pacientes com suspeita ou confirmação da doença^(16)^.

Em 12 de março de 2020, foram confirmados os primeiros casos importados e dois dias depois, foi estabelecida a transmissão local da COVID-19 no Recife. Nessa etapa, a vigilância municipal monitorava todas as pessoas notificadas^(16)^. Em 17 de março de 2020^(16)^, com o estabelecimento da transmissão comunitária e incremento no número de infectados, o monitoramento passou a ser apenas para as pessoas hospitalizadas, por meio de ligações telefônicas ou e-mail para familiar e/ou vigilância epidemiológica hospitalar (VEH). Diante da crescente demanda, o serviço municipal de vigilância epidemiológica foi reforçado gradativamente. Ocorreu a ampliação no número de profissionais envolvidos na vigilância dos vírus respiratórios, passando de três (dois técnicos e um coordenador), para 45 pessoas, destes, 36 enfermeiros.

### Coleta e organização dos dados

Os resultados descritos são provenientes da observação participante e estruturada, de pesquisadores, que atuaram na concepção, no desenvolvimento e na implementação da estratégia. Quatro pesquisadores são doutores com mais de 20 anos de experiência em saúde pública, vigilância e emergências em saúde pública, e uma pesquisadora é mestre, com experiência similar com menor tempo. Três pesquisadores atuam na Secretaria de Saúde do município, uma em universidade pública e uma em fundação de pesquisa social. Em relação àqueles com vínculo na secretaria, um atua no setor de atenção à saúde e os outros na vigilância epidemiológica e mortalidade, o que, por um lado, facilita o acesso às informações estratégicas para a reconstituição da intervenção e, por outro, pode influenciar a descrição das ocorrências e minimização dos obstáculos da gestão da pandemia.

A abordagem retrospectiva pode implicar em perda de informações, sobretudo pela não documentação de microprocessos de trabalho associados ao possível viés de memória. Para minimizar esses efeitos, o produto deste trabalho foi submetido aos técnicos envolvidos com a vigilância para confirmação ou não dos achados.

Além disso, realizou-se análise em documentos públicos relacionados a essa vigilância, disponíveis nos sites oficiais da Organização Mundial da Saúde, do Ministério da Saúde, da Secretaria de Saúde do Recife e de Pernambuco, como diretrizes internacionais, notas técnicas, manuais de codificação das causas de morte, de manejo de corpos durante a pandemia e plano de contingência para COVID-19 (Quadro 1). Os documentos foram acessados de agosto de 2022 a julho de 2023.

**Quadro 1:** Lista de documentos oficiais sobre a COVID-19 consultados da Organização Mundial da Saúde, do Ministério da Saúde, da Secretaria de Saúde de Pernambuco e do Recife, entre 2020 e 2021

Documento	Conteúdo
Diretrizes internacionais para a certificação e codificação da COVID-19 como causa de morte, Organização Mundial da Saúde, 20 de abril 2020^(17)^.	Apresenta a definição das causas de mortes, diretrizes para a certificação da COVID-19 como uma causa de morte e em termos de mortalidade.
Recomendações provisórias sobre gestão de cadáveres no contexto da COVID-19, Organização Mundial da Saúde, 7 de abril de 2020^(18)^.	Trata sobre gestão de cadáveres no contexto da COVID-19, autópsia, limpeza ambiental e equipamento de proteção individual.
Manual de manejo de corpos no contexto do coronavírus Sars-CoV-2 - COVID-19, versão 1 e 2, Ministério da Saúde, março e novembro de 2020^(19,20)^.	Aborda as recomendações para a realização de velórios, sepultamentos, necropsia e emissão de declaração de óbito (DO) no contexto da COVID-19.
Nota informativa n° 02 para codificação das causas de morte para óbitos suspeitos ou confirmados por COVID-19, março de 2020, Secretaria de Saúde de Pernambuco (SES-PE)^(21)^.	Versa sobre as recomendações para o preenchimento das causas de morte na PARTE I da DO, para óbitos suspeitos ou com resultado laboratorial positivo para COVID-19.
Nota técnica DG-IAEVE da SEVS/SES PE n° 04 de 25 de março de 2020, sobre manejo de corpos no contexto da infecção pelo novo coronavírus - COVID-19^(22)^.	Dispõe sobre o encaminhamento de corpos ao Serviço de Verificação de Óbito (SVO), Instituto Médico Legal (IML), às funerárias, realização de velório, sepultamento e orientação aos serviços de saúde sobre coleta de material para exames e preparo de corpos no contexto da COVID-19.
Nota técnica da SEVS da SES PE nº 7 de 14 de abril de 2020, sobre vigilância epidemiológica e laboratorial na pandemia da COVID-19^(23)^.	Atualiza os profissionais de saúde de Pernambuco sobre notificação, definição de caso suspeito, confirmado e inconclusivo de síndrome gripal (SG) e síndrome respiratória aguda grave (SRAG), coleta, acondicionamento e transporte de amostras e emissão de DO.
Nota informativa nº 07 de 05 de maio de 2021 da Gerência de Vigilância Epidemiológica da Secretaria de Saúde do Recife^(24)^.	Disponibiliza os objetivos, características, fluxo, atividades desenvolvidas e etapas da vigilância dos óbitos por COVID-19.
Plano Municipal de Contingência COVID-19 da Secretaria de Saúde do Recife (PE), 10 de março 2020^(16)^.	Descreve os níveis de resposta e atividades propostas, lista de áreas técnicas e responsáveis, fluxo assistencial de casos suspeitos na atenção primária à saúde e no Samu.

*Legenda: DO= declaração de óbito; VE= vigilância epidemiológica; VEH= vigilância epidemiológica hospitalar; SRAG= síndrome respiratória aguda grave; SIM= sistema de informação sobre mortalidade; SIVEP-Gripe= sistema de vigilância epidemiológica da gripe; Notifica PE=sistema de informação para registro de casos graves de COVID-19 utilizado em Pernambuco.*

### Etapas do trabalho

A pesquisa iniciou com a elaboração do modelo lógico, seus componentes e explicitação dos fluxos. Posteriormente, definiram-se as técnicas de coleta, a organização dos dados, foi construída a linha do tempo e as principais mudanças nos componentes da vigilância do óbito, considerando contextos nacionais e local. Por fim, apresentaram-se os achados da investigação dos óbitos no Recife (PE) de 2020 e 2021.

### Análise dos dados

Realizou-se relacionamento por meio de pesquisa automática das variáveis número da DO e nome do paciente da Planilha de Óbitos por SRAG e COVID-19 com o banco de dados do Sistema de Informação sobre Mortalidade (SIM) para os óbitos investigados e discutidos ocorridos em 2020 e 2021. Para cada DO emparelhada com a linha da Planilha de Óbitos, conferiu-se manualmente a data de nascimento e o nome da mãe para validação do par verdadeiro. Para os óbitos não localizados automaticamente, após pesquisa manual, todos foram localizados no SIM e foram analisadas as causas de óbitos, por meio do Microsoft Excel. O acesso aos dados secundários do SIM e da Planilha de Óbitos por SRAG e COVID-19, com informações nominais dos casos que evoluíram para óbito, foi autorizado pelo Comitê de Ética em Pesquisa da Universidade Federal de Pernambuco.

## RESULTADOS

A vigilância do óbito por COVID- 19 em Recife foi mediada, dinamicamente, a partir das características dessa emergência em saúde pública e dos determinantes socioeconômicos e de assistência à saúde nos contextos globais, do Brasil e de Recife. O desenvolvimento da vigilância do óbito foi ordenado gradativamente, devido às limitações impostas pelas medidas sanitárias vigentes e pela organização da vigilância municipal, a partir de seus componentes (Figura 1).

**Figura 1:** Modelo lógico, componentes operacionais e atividades da vigilância do óbito por COVID-19 no Recife, Pernambuco, Brasil, de 2020 e 2021

- Identificação dos óbitos por SRAG ou COVID-19.
- Investigação epidemiológica do óbito por SRAG ou COVID-19.
- Discussão dos óbitos por SRAG ou COVID-19.
- Conclusão da discussão e reclassificação dos óbitos por SRAG ou COVID-19.
- Qualificação dos sistemas de informação em saúde.
- Encaminhamento das recomendações aos gestores.

O componente inicial, identificação dos óbitos por SRAG ou COVID-19, objetivava captar os óbitos suspeitos ou confirmados de residentes no Recife, detectados por meio da notificação ou no monitoramento da evolução dos casos internados ou nas DO, quando descrito como SRAG ou COVID-19 nas causas de morte. O recolhimento das DO nos estabelecimentos de saúde, em especial com a inauguração de sete hospitais de campanha na cidade de Recife, passou a ser realizado com maior frequência, até três vezes semanais, quando comparado com rotina anterior à pandemia.

Entre 2020 e 2021, foram identificados 9.703 óbitos e inseridos na “Planilha de Óbitos por SRAG e COVID-19” pela equipe da vigilância epidemiológica municipal. O registro como SRAG dos óbitos suspeitos de infecção pela COVID-19 sem confirmação laboratorial seguia recomendação da Secretaria de Saúde de Pernambuco.

O componente subsequente, investigação dos óbitos por SRAG ou COVID-19, buscava a confirmação do endereço de residência e identificação dos eventos que contribuíram para o óbito. Devido à restrição sanitária, essas ações ocorriam por meio de ligação telefônica realizada pela equipe da vigilância epidemiológica municipal em 2020. Visava à identificação do endereço de residência correto, assistência prestada, complementação de informações, orientação ao familiar ou contato do caso que evoluiu para o óbito sobre medidas sanitárias, agendamento de exame e direcionamento ao atendimento psicológico quando necessário.

Os resultados de exames que complementavam a investigação eram obtidos por meio de consulta ao Gerenciador de Ambiente Laboratorial (GAL), sistema para laboratórios de saúde pública. Ocorrendo a não localização na busca inicial, solicitavam-se os resultados às equipes de VEH ou aos laboratórios privados.

Com a organização dos fluxos hospitalares, no final de 2020, passaram a ser adotados como critério de inclusão os óbitos ocorridos em serviços de saúde com VEH. A Secretaria de Saúde de Pernambuco elaborou ficha de investigação epidemiológica e estabeleceu a investigação dos óbitos por meio de consulta aos prontuários pela VEH. A ficha de investigação era composta por variáveis agrupadas em informações de identificação do caso; antecedentes pessoais; quadro clínico inicial e na hospitalização; diagnóstico na internação; achados laboratoriais e tomográficos; complicações; transporte pré-hospitalar; DO; classificação e critério de encerramento do óbito.

Nessa etapa, os óbitos podiam assumir desfechos, como: confirmado ou descartado para COVID-19, SRAG não especificada, outros vírus respiratórios, outras causas de óbito ou ser residente de outro município. Entre o total de óbitos registrados, 7,9% (764) corresponderam a residentes de outros municípios.

A discussão de óbitos, terceiro componente, destinava-se à identificação das possíveis falhas na assistência ocorrida nos serviços de saúde, reconhecendo a sua evitabilidade e contribuindo com o encerramento dos óbitos com menção de SRAG ou COVID-19. Essa fase possui função educativa, reflexiva e propositiva, e não coercitiva ou punitiva.

Em 2020, devido à limitação na oferta de exames laboratoriais específicos, foram eleitos para discussão todos os óbitos que não realizaram coleta de amostras biológicas. A partir do primeiro semestre de 2021, os critérios de seleção dos óbitos para a discussão passaram a ser: óbitos com resultado de exame positivo para COVID-19 e mais de 90 dias entre a data do início dos sintomas e a data do óbito, ou aqueles com intervalo inferior a 90 dias sem registro de COVID-19 ou SRAG na DO; 10% dos óbitos com resultado laboratorial inconclusivo e 5% com resultado negativo e 1% dos óbitos com resultado laboratorial positivo. Contudo, observou-se que, prioritariamente, discutiam-se os óbitos com investigações hospitalares disponíveis realizadas pelas equipes de VEH.

As discussões eram realizadas pelo grupo técnico-gestor da vigilância do óbito composto por profissionais da vigilância epidemiológica municipal, da área técnica de COVID-19, da mortalidade e por médicos da rede de saúde, pelo menos um infectologista. As reuniões eram semanais, eram discutidos até cinco óbitos, sempre de forma *on-line* ou híbrida na sede da vigilância. Alterava-se a periodicidade das reuniões devido à realocação dos profissionais da vigilância do óbito para outras atividades de acordo com as ondas de ocorrência de casos, ocorridas entre outubro de 2020 e janeiro de 2021 e entre março e junho de 2021. Foram investigados e discutidos, em grupo técnico, 89 óbitos ocorridos entre março de 2020 e dezembro de 2021, destes, 60 (67,4%) óbitos foram confirmados para COVID-19, 55 (61,8%) por critério laboratorial, 5 (5,6%) por clínico epidemiológico e 29 (32,6%) foram descartados.

O quarto componente, conclusão da discussão e reclassificação dos óbitos por SRAG ou COVID-19, buscava identificar as possíveis falhas na assistência associadas aos óbitos e definia as recomendações de intervenção a serem encaminhadas. Os óbitos eram finalizados com as informações obtidas na investigação em: confirmado laboratorialmente, clinicamente ou por critério epidemiológico e descartado.

No primeiro ano da pandemia, para fins de registro nos sistemas de informação de notificação de casos, Notifica PE e Sivep-Gripe, era necessário o resultado laboratorial confirmando ou descartando o óbito. Apesar de constar em notas técnicas a possibilidade do encerramento do caso de COVID-19 pelo critério clínico epidemiológico, utilizava-se a classificação SRAG não especificada para aqueles sem coleta de amostras laboratoriais.

No que se refere ao registro dos óbitos de COVID-19, em março de 2020, o Ministério da Saúde e a Secretaria de Saúde de Pernambuco recomendaram a utilização do código U04.9 (SRAG) para os óbitos com suspeita de infecção por COVID-19 sem diagnóstico laboratorial. Para aqueles com resultado laboratorial positivo para COVID-19, preenchia-se a parte I da DO com código B34.2 (infecção por coronavírus - COVID-19) e para os óbitos com doença respiratória aguda devido ao COVID-19, utilizava-se o marcador, código U04.9, na mesma linha que o código B34.2.

O componente da qualificação dos Sistemas de Informação em Saúde (SIS) tinha o propósito de corrigir as estatísticas oficiais. Após a conclusão do óbito, procedia-se a requalificação das causas de morte no SIM conforme recomendação da Organização Mundial da Saúde em confirmado laboratorialmente código B34.2 + U07.1 da Classificação Estatística Internacional de Doenças e Problemas Relacionados à Saúde (CID-10), confirmado clínico epidemiológico código CID-10 B34.2 + U07.2 e descartado com a utilização do marcador código CID-10 U04.9^(17)^. As codificações das causas de morte do antes e depois das discussões eram realizadas por codificadores de causa básica treinados, com ampla experiência, atuantes na Secretaria Municipal de Saúde.

Os códigos da CID-10, principalmente da Parte II da DO, referente às morbidades pré-existentes e sem relação direta com a morte, seguido da linha B, sofreram modificações. A causa básica apresentou 34,8% de alteração após a discussão e qualificação dos óbitos (Tabela 1).

**Tabela 1:** Distribuição do número e proporção de óbitos com manutenção ou alteração do código CID-10 após a discussão, segundo sequência das causas de morte. Recife, Pernambuco, Brasil, 2020-2021

		Óbitos com manutenção do Código da CID-10		Óbitos com alteração do Código da CID-10	
		n°	%	n°	%
PARTE I	Linha A	49	55,1	40	44,9
	Linha B	38	42,7	51	57,3
	Linha C	50	56,2	39	43,8
	Linha D	74	83,1	15	16,9
PARTE II		25	28,1	64	71,9
CAUSA BÁSICA		58	65,2	31	34,8

*Nota: CID-10= Classificação Estatística Internacional de Doenças e Problemas Relacionados à Saúde.*

Em relação à análise exclusivamente da causa básica de morte, dos 89 óbitos investigados e discutidos no período de 2020 e 2021, 64 (71,9%) tiveram a COVID-19 como causa básica, após a conclusão do caso, houve a reclassificação de 14 (15,7%) óbitos para outras causas. Para aqueles óbitos (25; 28,1%) que não possuíam a COVID-19 como causa básica, seis (6,7%) sofreram a qualificação e foi atribuída a COVID-19 como causa determinante da morte.

O último componente, encaminhamento das recomendações aos gestores, tinha como objetivo enviar os relatórios dos óbitos investigados e discutidos aos responsáveis pelos setores de vigilância, assistência, regulação e planejamento em saúde municipal. Após a discussão, era elaborado um relatório de cada óbito, encaminhado aos gestores para a tomada de decisões, contendo: os problemas identificados e a requalificação das causas de morte. Esse procedimento foi o mais escasso, realizado para os óbitos discutidos nas primeiras reuniões.

Dos 89 óbitos discutidos, 52 (58,4%) tiveram falhas identificadas pelo grupo técnico durante o processo de adoecimento e morte, ao passo que para 37 (41,5%) óbitos, não houve esse apontamento. Para os óbitos em que se identificaram falhas, suas respectivas recomendações foram agrupadas e relacionadas ao diagnóstico, de cunho individual, assistencial, de registro e notificação (Quadro 2).

**Quadro 2:** Principais falhas e recomendações identificadas na discussão de óbitos. Recife, Pernambuco, Brasil, 2020-2021

Tipo	Falha	Recomendação
Diagnóstico	Ausência de solicitação e/ou realização de exames laboratoriais clínicos e de imagens Realização tardia de exame de Reação da Transcriptase Reversa com Reação em Cadeia da Polimerase (RT - PCR) para COVID-19	Realização oportuna do exame de RT-PCR para COVID-19 Realização de exames de imagem para auxílio no diagnóstico
Individual	Busca tardia à assistência à saúde Ausência de esquema vacinal completo para COVID-19	Otimização da vinculação entre a equipe da atenção primária e os usuários do território Promoção de ações educativas sobre sinais de alerta de gravidade da doença Realização de busca ativa para entendimento da falta de esquema vacinal para COVID-19
Assistencial	Não realização de avaliação cardiológica Transferência indevida para hospital de referência para COVID-19 de paciente com resultado negativo de RT - PCR Ausência de cuidados gerais de enfermagem Falta de medicamentos sedativos e broncodilatadores Falta ou problema no funcionamento de equipamentos hospitalares (ventiladores mecânicos, bomba de infusão, fonte de oxigênio, aquecedor) Falha no acesso aos serviços de saúde (ausência de vaga em leito de UTI) Iatrogenia relacionada à assistência médica Infecção relacionada à assistência à saúde (COVID-19, infecção urinária fúngica) Falha na vigilância e segurança do paciente	Presença de supervisão de enfermagem nos serviços hospitalares Disponibilização de equipamentos e medicamentos para assistência ao paciente Elaboração de protocolos e treinamento para boas práticas em procedimentos invasivos Avaliação e reorganização dos fluxos assistenciais (área limpa e área contaminada) Aplicação do protocolo de segurança do paciente nos serviços Aplicação do protocolo de prevenção de infecção nosocomial nos serviços Disponibilização de equipe de manutenção de equipamentos Avaliação crítica e especializada do quadro clínico para transferência oportuna
Registro e Notificação	Falha no detalhamento ou ausência de registros intra-hospitalar e para transferência do paciente Divergência de informações entre as diversas fontes de registros	Registro detalhado em prontuários hospitalares e em resumos de transferência Preenchimento completo da ficha de investigação de óbitos de SRAG

*Nota: UTI = Unidade de Terapia Intensiva; RT-PCR= reação da transcriptase reversa seguida pela reação em cadeia da polimerase; SRAG= síndrome respiratória aguda grave.*

Os principais obstáculos na execução dos componentes da vigilância do óbito estavam relacionados às poucas evidências clínicas sobre a doença, à oferta limitada de testes diagnósticos, à ausência de códigos da CID-10 para a COVID-19, ao acesso restrito aos serviços de saúde e ao manuseio de prontuários no início da pandemia. Do mesmo modo, a realocação de profissionais para atividades não relacionadas à vigilância do óbito foi outro entrave ao seu pleno desenvolvimento (Quadro 3).

**Quadro 3:** Facilitadores e obstáculos segundo componente da vigilância do óbito por COVID-19. Recife, Pernambuco, Brasil, 2020-2021

Componente	Facilitadores	Obstáculos
Identificação dos óbitos por SRAG ou COVID-19	Monitoramento da evolução dos casos internados, identificando os óbitos	Evidências clínicas sobre a doença limitada no início da pandemia
Investigação epidemiológica do óbito por SRAG ou COVID-19	Experiência da equipe de vigilância epidemiológica municipal e hospitalar em investigações de casos e óbitos de doenças de notificação compulsória	Restrição de acesso aos serviços de saúde e de manuseio de prontuários no início da pandemia
Discussão dos óbitos por SRAG ou COVID-19	Disponibilidade de equipamentos de videoconferência para realização de reuniões de discussão de óbitos *on-line*	Oferta de testes diagnósticos limitados Realocação de profissionais da vigilância do óbito
Qualificação dos sistemas de informação em saúde	Qualificação dos sistemas de informações incorporada previamente à rotina da vigilância epidemiológica municipal	Mudança de definição de caso da doença Ausência de códigos da CID-10 para a COVID-19

*Nota: SRAG= síndrome respiratória aguda grave. CID-10= Classificação Estatística Internacional de Doenças e Problemas Relacionados à Saúde.*

## DISCUSSÃO

A análise da transformação da vigilância do óbito por COVID-19 durante a emergência sanitária mostrou que a intervenção foi desenvolvida para evitar novos óbitos decorrentes de situações semelhantes, entender a cadeia de eventos e qualificação dos SIS. Iniciou a partir da notificação das primeiras mortes pelos serviços de saúde à vigilância epidemiológica municipal, envolveu os setores responsáveis pela vigilância epidemiológica das doenças transmissíveis, pelo Centro de Informações Estratégicas de Vigilância em Saúde (Cievs) e pela mortalidade.

Todos os componentes foram gradativamente operacionalizados pela vigilância epidemiológica do Recife. Inicialmente, a vigilância do óbito foi desenvolvida para resgatar informações (endereço, história clínica e resultados laboratoriais), qualificar os SIS e prestar orientações aos contatos e/ou familiares das pessoas que faleceram. Posteriormente, incrementou-se, com a realização da investigação hospitalar, discussão dos óbitos, identificação dos problemas e proposições de recomendações aos gestores.

Estudos evidenciam que houve uma determinação social na incidência e na mortalidade por COVID-19. A expansão da pandemia ocorreu para áreas de maior vulnerabilidade, associadas à crise econômica, fatores socioeconômicos, condições de moradia inadequada, maior proporção de pessoas vivendo em moradias com adensamento excessivo e maior percentual de pessoas sem escolaridade^(25)^.

A captação dos óbitos realizada pela vigilância epidemiológica, além de COVID-19, incluía SRAG entre as causas registradas na DO, para abranger casos sem confirmação laboratorial. Inclusão alinhada com recomendação da Organização Mundial da Saúde para o monitoramento das mortes relacionadas a causas respiratórias não específicas, vinculadas a poucas certezas acerca da doença e variabilidade da oferta de testes^(26,27)^. A subnotificação de óbitos foi evidenciada em estudo no continente africano, de vigilância pós-morte, com a realização RT-PCR nos falecidos^(8)^.

A confirmação do endereço de residência, parte da investigação inicial dos casos, permitiu retificar essa informação, aproximando o número real de mortes de residentes. Sabe-se que a completitude das fichas de investigação fornece informações, direciona intervenções e investimentos^(28)^. Observa-se limitações na investigação de óbitos infantis e fetais, devido à dificuldade na validação do endereço de residência do falecido ou da mãe^(29)^.

Os resultados de exames específicos eram resgatados em diversas fontes, durante a investigação dos óbitos. O reconhecimento das pessoas infectadas permite o seu isolamento oportuno e adequado^(30)^. Todavia, no início da pandemia, a abrangência da testagem era limitada, especialmente em países de baixa renda ou em desenvolvimento, em decorrência do alto custo dos insumos e mão de obra^(30)^. No Recife, os insumos para a coleta e o processamento da amostra eram de responsabilidade da Secretaria Estatual de Saúde, ficando para o município, a realização da coleta e disponibilização do resultado ao paciente.

Os óbitos ocorridos em unidades de saúde sem VEH não foram eleitos para a discussão, devido às medidas sanitárias que restringiam o acesso aos serviços de saúde e prontuários. A VEH exerce papel relevante na unidade notificadora, integrando sistemas de informação e sensibilizando profissionais, durante resposta às outras emergências em saúde pública de interesse nacional e internacional, como na pandemia de influenza A HIN1 e na de microcefalia relacionada ao Zika vírus^(31)^. Destaca-se a atuação do enfermeiro na VEH e na vigilância epidemiológica municipal. Esse profissional, além da atuação na assistência clínica, dentro do contexto da pandemia, possui habilitação, competência técnica e legal no desenvolvimento de investigação e comunicação^(32)^.

Devido à dinamicidade do contexto de uma Espii, a periodicidade das reuniões de discussão foi modificada e as recomendações aos gestores não foram encaminhadas para todos os óbitos. Apesar da contratação e mobilidade de profissionais, sobretudo enfermeiros, de outros setores para vigilância epidemiológica, ocorreu o reposicionamento interno para atividades diferentes das relacionadas à vigilância do óbito. A atuação desses profissionais foi redirecionada para orientação aos familiares e correção das causas de morte. A disseminação de notícias falsas sobre a origem, os sintomas e o medicamento para a COVID-19, potencializada no Brasil pela negação do governo federal à relevância da pandemia e às medidas de distanciamento, conduziu para a necessidade de orientação contínua à população^(33,34)^.

Ainda que, com o redirecionamento das equipes de vigilância epidemiológica municipal, observou-se a qualificação das causas de morte, com a modificação do código da CID-10, da Parte II da DO dos óbitos investigados e discutidos. Esse achado encontra embasamento no estudo sobre a qualidade dos registros dos óbitos por COVID-19 entre março de 2020 e dezembro de 2021, que identificou a manutenção da qualidade das informações do SIM no Recife por meio da análise da completitude, durante a pandemia quando comparado a anos anteriores^(35)^.

A contribuição da vigilância dos óbitos na qualificação das causas de morte, incluindo a COVID-19 como causa básica, corrobora estudo realizado, no primeiro ano da pandemia em países africanos, em que a análise dos óbitos melhorou as estimativas de mortalidade pela doença^(8)^. Na cidade de Nova York, a inspeção dos atestados contribuiu para números mais reais de mortes de COVID-19 em 2020^(26)^. Na Irlanda, em 2022, a vigilância do óbito auxiliou no registro adequado da causa de morte relacionada à COVID-19^(9)^.

A vigilância do óbito por COVID-19, instituída durante uma Espii, permanece ativa no Recife, com adaptação dos critérios de seleção dos óbitos a serem discutidos, frente ao cenário endêmico da COVID-19. Além da COVID-19, as mortes infantis, maternas e por tuberculose são objeto de vigilância de óbito no Recife^(7,35-37)^.

O estudo permitiu mostrar a importância da vigilância do óbito para no Recife, ao usá-la como estratégia para reduzir a subnotificação, qualificar as estatísticas de mortalidade e recomendar medidas de promoção, prevenção, diagnóstico e tratamento para evitar novos óbitos. Ainda que a experiência seja recente e com necessidade de aperfeiçoamento, a partir das lições aprendidas durante a Espii, acredita-se no seu potencial para sua utilização nacionalmente, uma vez que se baseou nas vigilâncias de óbito consolidadas no Brasil, como a infantil, fetal e materno.

Os achados reforçam a importância de iniciativas que fortaleçam e promovam avanço da vigilância de óbito. O desenvolvimento da vigilância do óbito por COVID-19 teve como limitador a dificuldade de acesso a exames diagnósticos, subnotificação, picos de ocorrência da doença e reposicionamento de profissionais para outras atividades.

### Limitações do Estudo

O estudo possui como limitação a indisponibilidade de documentos ou outros tipos de fontes de informação em que fosse possível identificar fluxos da vigilância epidemiológica municipal e registro de datas precisas do momento das mudanças de condutas e rotinas para a vigilância do óbito. Outrossim, a avaliação da transformação da intervenção, ao usar uma abordagem retrospectiva é passível do viés de memória o que pode implicar em perda de informações. Por outro lado, ao se basear em uma concepção dinâmica da vigilância do óbito por COVID-19, na vigência de um período pandêmico, a fronteira entre a intervenção e seu contexto é tênue, aspecto que pode contribuir para submergir aspectos contextuais relevantes não aprofundados no desenho. Nessa situação, destaca-se que o número de variáveis contextuais importantes excedeu muito os pontos de observação.

### Contribuições para as áreas de Enfermagem, Saúde e Políticas Públicas

A vigilância do óbito por COVID-19 desenvolveu-se durante a maior Espii, um cenário desafiador e de incertezas. Apesar da alta rotatividade de profissionais, agudizados pela pandemia e decorrente do frequente remanejamento de funções, a vigilância do óbito contribuiu para a melhoria da qualidade da informação, adoção de medidas preventivas e redução da mortalidade. Outrossim, desempenha função formativa para enfermeiros e demais profissionais envolvidos nessa vigilância nas secretarias de saúde e equipes de VEH. Ademais, poucos trabalhos que analisem a transformação da intervenção durante as mudanças impostas pela Espii estão disponíveis e a sua contribuição para a vigilância do óbito por COVID-19 demonstra a sua relevância.

## CONSIDERAÇÕES FINAIS

A análise da transformação da vigilância do óbito por COVID-19 mostrou as adaptações dos componentes da intervenção no contexto em que foi desenvolvida, imposta pela emergência em saúde pública e contribuiu para a melhoria na especificação das causas de morte. O remanejamento de profissionais afetou a periodicidade das discussões dos óbitos e o encaminhamento das recomendações aos gestores sobre os problemas identificados. Embora efetuada durante a Emergência em Saúde Pública de Interesse Internacional, a vigilância do óbito por COVID-19 revelou-se uma importante estratégia, contribuindo com a qualificação da informação, o planejamento e a organização dos fluxos assistências e de vigilância, bem como a recomendação de medidas preventivas para a redução da mortalidade.
